# Effects of Mild Closed-Head Injury and Subanesthetic Ketamine Infusion on Microglia, Axonal Injury, and Synaptic Density in Sprague–Dawley Rats

**DOI:** 10.3390/ijms25084287

**Published:** 2024-04-12

**Authors:** Martin Boese, Rina Y. Berman, Jennifer Qiu, Haley F. Spencer, Kennett D. Radford, Kwang H. Choi

**Affiliations:** 1 Daniel K. Inouye Graduate School of Nursing, Uniformed Services University, Bethesda, MD 20814, USA; martin.boese@usuhs.edu (M.B.); kennett.d.radford.mil@health.mil (K.D.R.); 2 Center for the Study of Traumatic Stress, Uniformed Services University, Bethesda, MD 20814, USA; rina.berman.ctr@usuhs.edu; 3 Department of Chemistry and Biochemistry, University of Maryland, College Park, MD 20742, USA; jenniferqiu265@gmail.com; 4 Program in Neuroscience, Uniformed Services University, Bethesda, MD 20814, USA; haleyspencer121@gmail.com; 5 Department of Psychiatry, F. E. Hébert School of Medicine, Uniformed Services University, Bethesda, MD 20814, USA

**Keywords:** mild traumatic brain injury, ketamine, axonal injury, lateral geniculate nucleus, synaptic density, rats

## Abstract

Mild traumatic brain injury (mTBI) affects millions of people in the U.S. Approximately 20–30% of those individuals develop adverse symptoms lasting at least 3 months. In a rat mTBI study, the closed-head impact model of engineered rotational acceleration (CHIMERA) produced significant axonal injury in the optic tract (OT), indicating white-matter damage. Because retinal ganglion cells project to the lateral geniculate nucleus (LGN) in the thalamus through the OT, we hypothesized that synaptic density may be reduced in the LGN of rats following CHIMERA injury. A modified SEQUIN (synaptic evaluation and quantification by imaging nanostructure) method, combined with immunofluorescent double-labeling of pre-synaptic (synapsin) and post-synaptic (PSD-95) markers, was used to quantify synaptic density in the LGN. Microglial activation at the CHIMERA injury site was determined using Iba-1 immunohistochemistry. Additionally, the effects of ketamine, a potential neuroprotective drug, were evaluated in CHIMERA-induced mTBI. A single-session repetitive (ssr-) CHIMERA (3 impacts, 1.5 joule/impact) produced mild effects on microglial activation at the injury site, which was significantly enhanced by post-injury intravenous ketamine (10 mg/kg) infusion. However, ssr-CHIMERA did not alter synaptic density in the LGN, although ketamine produced a trend of reduction in synaptic density at post-injury day 4. Further research is necessary to characterize the effects of ssr-CHIMERA and subanesthetic doses of intravenous ketamine on different brain regions and multiple time points post-injury. The current study demonstrates the utility of the ssr-CHIMERA as a rodent model of mTBI, which researchers can use to identify biological mechanisms of mTBI and to develop improved treatment strategies for individuals suffering from head trauma.

## 1. Introduction

Traumatic brain injury (TBI) is a leading cause of death and disability [1]. Mild TBI (mTBI) is the most common form of TBI, accounting for approximately 80% of reported cases. Although most people fully recover from mTBI, a subset of patients experience persistent symptoms [2,3]. These post-concussive symptoms include emotional disturbances, cognitive impairments, and physical symptoms including visual disturbances [4]. The mTBI consists of primary injury (effects directly tied to the mechanical forces at the moment of impact) and secondary injury (biochemical processes occurring in the hours or days following primary injury) [5,6]. The consequences of primary injury such as axonal damage are generally regarded as irreversible, but secondary injury such as neuroinflammatory processes may be a target for mTBI treatment [5]. Ketamine, a multimodal drug used in traumatically injured patients, has anti-inflammatory [7] and synaptogenic effects [8] and may even reverse synaptic deficits induced by TBI [9]. However, very few preclinical studies have examined the utility of intravenous (IV) ketamine infusion in ameliorating or preventing secondary injury after mTBI.

There is a major gap between preclinical and clinical mTBI studies. A majority of preclinical mTBI studies use open-head injury models, requiring craniotomy and direct impact to the brain, whereas human mTBIs often occur as blunt trauma to an intact skull (closed-head injury) or blast injury [10]. In addition, many preclinical TBI paradigms utilize the stereotactic frame, which restrains the subject’s head when delivering impacts. A recently developed paradigm, the closed-head impact model of engineered rotational acceleration (CHIMERA), simulates the mechanics of a closed-head injury occurring in humans by impacting an unrestrained head, allowing it to rotate freely [11]. This is critical to model a clinically relevant mTBI, as the human brain experiences significantly higher strain forces (deformation) in response to rotational acceleration than linear acceleration [12]. Post-concussive symptoms observed after mTBI fall into cognitive, physical, and emotional deficits [4]. A mild CHIMERA paradigm induced working and spatial memory deficits, balance problems, and anxiety in mice [11]. Human mTBI is also marked by cytokine changes [13], white-matter damage [14], and microgliosis [15], all of which are observed after mild CHIMERA [11,16,17]. Notably, this CHIMERA paradigm does not induce major tissue loss or structural damage, confirming the mild nature of the injury [11,17]. CHIMERA also produces similar head kinematics to those observed in human sports-related concussions [11]. Therefore, we utilized the CHIMERA as a rodent mTBI model in the current study.

Ketamine, a non-competitive N-methyl-D-aspartate receptor (NMDAR) antagonist, is used as a multi-modal trauma analgesic because it does not produce respiratory depression or cardiovascular instability at sub-anesthetic doses [18,19,20]. The multi-modal properties of ketamine, including potential anti-inflammatory effects [7], may be beneficial for the treatment of TBI. Ketamine has been shown to be neuroprotective in preclinical models of post-traumatic stress disorder (PTSD), major depression, and TBI [9,21,22,23,24]. One study reported that a daily ketamine injection (10 mg/kg, intraperitoneal (IP)) increased dendritic arborization, length, and spine density in the hippocampus after a weight-drop-induced moderate TBI in rats [9]. However, no studies to date have examined synaptic density following a clinically relevant IV ketamine infusion after mTBI. We have established a clinically relevant paradigm of IV ketamine infusion in awake and freely moving rats [25,26] and combined this technique with CHIMERA to investigate the effects of mTBI and ketamine on inflammatory cytokines and behavioral outcomes [7]. Though IV ketamine infusion reduced certain inflammatory cytokines regardless of the injury condition, its effects on other injury outcomes, such as microglial morphology and synaptic density, are largely unknown.

Microglia, the primary immune cells in the brain, are critically important in TBI and serve roles such as initiating neuroimmune responses, clearing cellular debris, and releasing numerous substances that may have beneficial or harmful effects depending on biological context [27,28]. Microglial activation is typically measured via the upregulation of microglial biomarkers after mTBI [15,16,29]. However, this analysis is incomplete as microglia have important morphological states corresponding to inflammatory activation or quiescence. Microglia at rest are referred to as ramified, having numerous long, branching processes; in contrast, activated microglia are marked by several morphological states including hypertrophied with extended processes and bushy with an enlarged cell body and shorter, thicker branches [30]. At the final stage of activation, microglia become ameboid, with a rounded cell body and few to no processes [30]. Numerous rodent studies have found that morphologically active microglia are upregulated after mTBI [31,32,33]. Ketamine has immunomodulatory properties, which include suppression of microglial activity [34]. However, the effects of IV ketamine on microglial morphology after mTBI have not been studied.

White-matter damage, particularly after injuries involving rapid acceleration and deceleration, is a hallmark of TBI and may be implicated in consequences such as loss of consciousness [35,36]. This shearing of axons can lead to diffuse axonal injury (DAI), marked by axonal swelling, impaired axonal transport, and eventual disconnection of the axon [35]. Numerous symptoms have been attributed to white-matter damage. A subset of veterans with mTBI experienced decrements in executive function concurrent with loss of integrity in the prefrontal white matter, corpus callosum, and cingulum bundles [37]. After blast mTBI, the number of regions with white-matter damage is related to cognitive impairment [38] and physical post-concussive symptoms [39]. Furthermore, loss of white-matter integrity in several regions was correlated with worse outcome as measured by the Glasgow Outcome Scale-Extended [40]. As such, white-matter damage after mTBI may be functionally relevant. 

Similar to humans, white-matter damage can be observed in rodents after mTBI. Axons in white-matter tracts are particularly vulnerable to injury due to their long and thin geometry, as well as to the tendency to become stiff and brittle under accelerative force [35]. The optic tract (OT), a continuation of the optic nerve, may be particularly vulnerable to TBI, as its multiplanar geometry in rodents [41] may introduce ample opportunities for impaction, shearing, and stretch injuries. Axonal injury in the OT is well established in rodent mTBI studies [7,11,16,17,42,43] and can be accompanied by impaired visual acuity and visual function [42,43]. Repetitive mTBI (rmTBI) using the CHIMERA paradigm over multiple injury days produced axonal injury in the OT of rodents [11,17,42,43]. Moreover, repetitive mTBI in a single session also produced axonal injury in the OT of rats [7]. These results suggest that mTBI produces white-matter damage such as axonal injury in the visual system of rodents.

Axons from the retinal ganglion cells (RGCs) form the optic nerves, which cross at the optic chiasm and continue as the OTs. The OT synapses onto the lateral geniculate nucleus (LGN), a visual relay center in the thalamus that is reciprocally innervated with the primary visual cortex [44]. Both the OT and LGN experience degenerative damage after TBI, corroborating vulnerability of the visual system following brain injury [45,46]. The visual system presents an intriguing pathway to examine synaptic plasticity after brain injury because it can produce trans-synaptic neurodegeneration across multiple brain regions [47]. Previous studies have quantified synaptic density in the brain after mTBI using dendritic branching, spine density, and levels of synaptic proteins [48,49,50]. Interestingly, one study found that the repeated weight-drop mTBI did not produce synaptic density changes in the LGN as measured by synaptophysin, a marker for pre-synaptic vesicles [51]. However, the methods used in those studies have some limitations. Manual counting of dendritic spines after imaging with electron microscopy is labor-intensive. Additionally, synaptic proteins are typically quantified using Western blot analysis, which does not provide spatial resolution information between pre- and post-synaptic puncta. A novel method to quantify synaptic density in the brain is the synaptic evaluation and quantification by imaging nanostructure (SEQUIN), which quantifies synaptic puncta double-labeled against presynaptic synapsin and post-synaptic density 95 (PSD-95) [52,53]. Synapses are defined as pairs of collocated pre- and post-synaptic puncta based on super-resolution confocal microscopy [52,53]. Thus, SEQUIN offers quantifiable spatial resolution of synaptic puncta, with a high-throughput data analysis workflow as compared to conventional methods such as dendritic analysis with Golgi staining or electron microscopy. The current study utilized the SEQUIN method to determine synaptic density in the LGN of rats that sustained ssr-CHIMERA injury.

The overall goal of this study was to characterize ssr-CHIMERA as a clinically relevant mTBI model and test ketamine, a potential anti-inflammatory drug, on neuroinflammation and synaptic plasticity in rats. We hypothesized that ssr-CHIMERA may increase microglial activation and axonal injury and decrease synaptic density in the brain, and ketamine infusion may ameliorate those effects. Additionally, we aimed to validate the new SEQUIN workflow for efficient analysis of brain synaptic plasticity following mTBI and a pharmacological intervention.

## 2. Results

**Figure 1:**
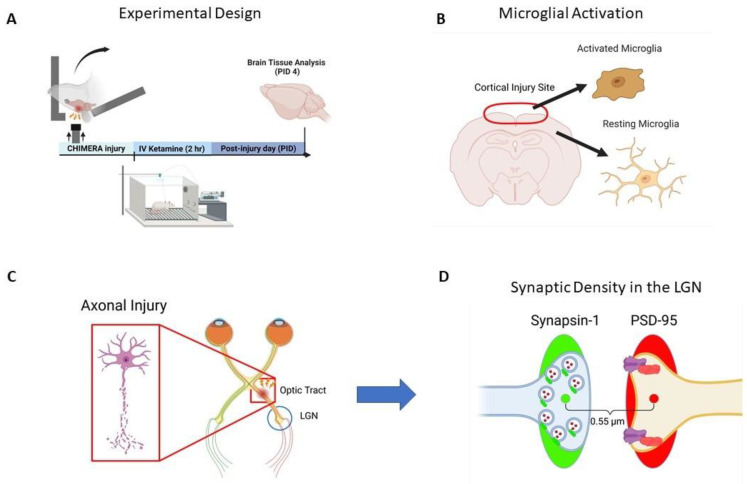
The overall study design and brain tissue analysis. (**A**): The timeline of the experiment including ssr-CHIMERA, IV ketamine infusion, and brain tissue collection for microglia, axonal injury, and synaptic density analyses. (**B**): CHIMERA injury site on the cerebral cortex (above the bregma) and examples of activated and inactive microglia. (**C**): Illustration of axonal injury in the OT (white-matter damage). (**D**): Synaptic density analysis using SEQUIN with pre-synaptic (synapsin) and post-synaptic (PSD-95) markers, shown as spots (ovals) and puncta (small circles). Synapses are defined as a pair of pre- and post-synaptic puncta separated by <0.55 μm.

Figure 1A shows the timeline of the experiment including ssr-CHIMERA, IV ketamine infusion, and brain tissue collection for immunohistochemistry. Brain tissue samples were collected at post-injury day 4 (PID-4) for silver staining, Iba-1 immunohistochemistry, and synaptic density analysis. Figure 1B depicts a CHIMERA injury site above the bregma and microglial activation at the injury site. Axonal injury in the OT was determined using silver staining as shown in Figure 1C. The RGCs project to the LGN via the OT. Figure 1D describes the SEQUIN method to determine synaptic density using immunofluorescent double-labeling of presynaptic (synapsin) and postsynaptic (PSD-95) markers with super-resolution confocal microscopy and efficient data analysis workflow.

**Figure 2:**
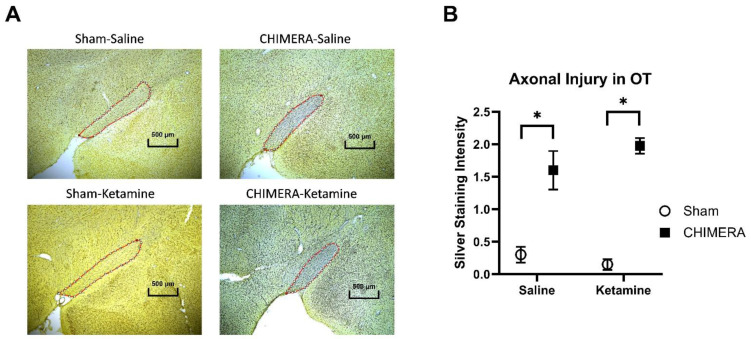
Axonal injury in the OT following ssr-CHIMERA injury in rats. (**A**): Representative of silver staining images in the OT (stereotaxic coordinates, AP: −2.92 mm, ML: ±3.4 mm, DV: 8.4 mm from the bregma), taken at 4× magnification. (**B**): CHIMERA injury significantly increased axonal injury in the OT compared to the sham group (N = 10 per group, * *p* < 0.05). Reproduced from the previous study with permission [7].

The effects of ssr-CHIMERA on axonal injury in the OT are shown in Figure 2. Representative images of silver staining in the OT are shown in Figure 2A, including sham-saline, CHIMERA-saline, CHIMERA-ketamine, and sham-ketamine in a clockwise direction. Figure 2B shows the significant effects of CHIMERA injury on silver staining intensity as compared to the sham injury (*p* < 0.001), indicating increased axonal injury in the white matter.

**Figure 3:**
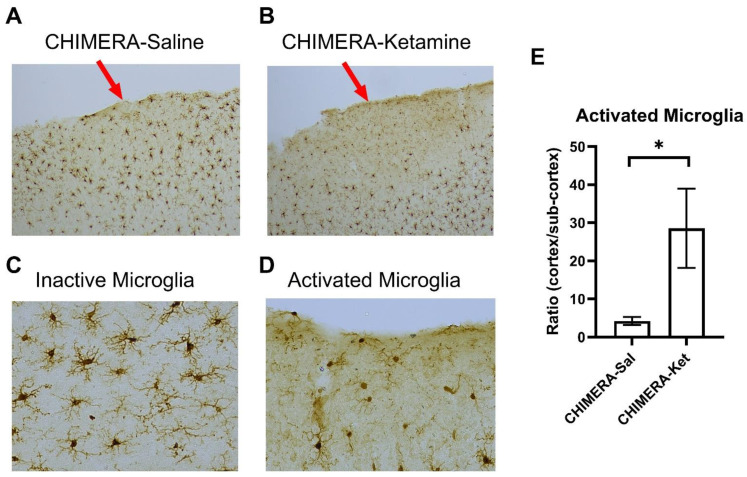
Microglial activation on the cortical injury site (primary motor cortex and somatosensory cortex) following ssr-CHIMERA in rats. (**A**): A representative image of the cortical injury site (arrow) in a CHIMERA-saline animal (10×). (**B**): A representative image of cortical injury site (arrow) in a CHIMERA-ketamine animal (10×). (**C**): A representative image of inactive (ramified) microglia (40×). (**D**): A representative image of activated (ameboid) microglia (40×). (**E**): Increased microglial activation in the CHIMERA-ketamine group compared to the CHIMERA-saline group (N = 6 per group, * *p* < 0.05).

The effects of ssr-CHIMERA and ketamine infusion on microglial activation at the injury site (primary somatosensory cortex) were determined using Iba-1 immunohistochemistry (Figure 3). Figure 3A shows the impact site in a CHIMERA-saline animal with mild microglial activation. However, an IV ketamine infusion (10 mg/kg) after ssr-CHIMERA injury produced a robust increase in microglial activation at the injury site (Figure 3B). Representative images of inactive (ramified) microglia and activated (ameboid) microglia are shown in Figure 3C and Figure 3D, respectively. The ratios of activated microglia between the cortical injury site and sub-cortical non-injury site were significantly different between the CHIMERA-saline and the CHIMERA-ketamine groups (t = 2.326, df = 10, *p* = 0.042) as shown in Figure 3E.

**Figure 4:**
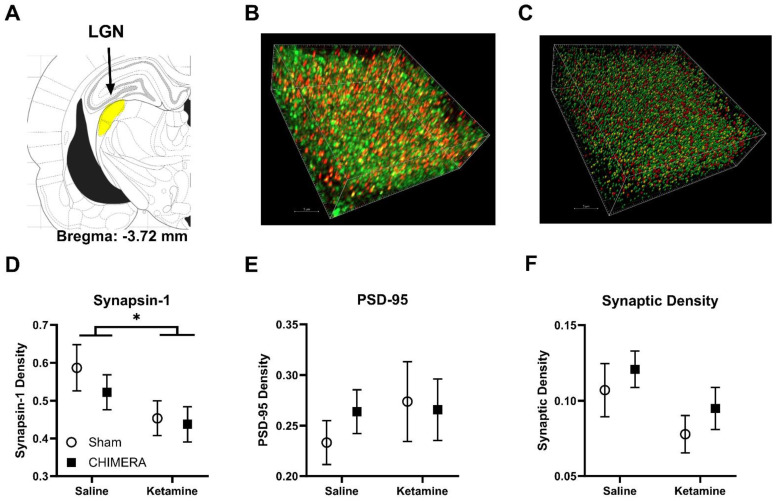
The SEQUIN analysis for synaptic density in the LGN. (**A**): The LGN (in yellow) depicted in the rat brain atlas (AP: −3.72 mm, ML: ±3.5 mm, DV: 4.6 mm from the bregma). (**B**): A confocal microscope image of synapsin (green), PSD-95 (red), and synaptic puncta (yellow) in the LGN. (**C**): A processed image of spot analysis using Imaris software version 10.0.1. (**D**): Ketamine infusion reduced synapsin density in the LGN. (**E**): No significant effects of CHIMERA or ketamine on PSD-95 density in the LGN. (**F**): No significant effects of CHIMERA or ketamine on synaptic density in the LGN. (N = 9–10 per group, * *p* < 0.05).

Figure 4 describes the SEQUIN results on synaptic density in the LGN following ssr-CHIMERA and ketamine infusion in rats. The LGN, located below the hippocampus, is shown in yellow in the rat brain atlas (Figure 4A). Representative confocal microscope images of synapsin (green), PSD-95 (red), and synaptic puncta (yellow) are shown in Figure 4B (unprocessed raw image) and Figure 4C (processed image with spot analysis). A two-way ANOVA revealed a main effect of ketamine (F_[1, 33]_ = 4.824, *p* = 0.035) on synapsin density (Figure 4D). There was no interaction between CHIMERA and ketamine on synapsin density (F_[1, 33]_ = 0.2416, *p* = 0.626). A two-way ANOVA revealed no significant effects of CHIMERA (F_[1, 33]_ = 0.1430, *p* = 0.708) or ketamine (F_[1, 33]_ = 0.5032, *p* = 0.483) or interaction on PSD-95 density (F_[1, 33]_ = 0.4144, *p* = 0.524) (Figure 4E). A two-way ANOVA on synaptic density indicated a trend toward significance on ketamine (F_[1, 33]_ = 3.798, *p* = 0.059) but not CHIMERA (F_[1, 33]_ = 1.193, *p* = 0.283) as shown in Figure 4F. These results indicate no significant effects of either CHIMERA injury or ketamine on synaptic density in the LGN at PID-4.

## 3. Discussion

The ssr-CHIMERA produced mild TBI phenotypes including microglial activation at the injury site and axonal injury in the optic tract. A subanesthetic IV ketamine infusion (10 mg/kg) enhanced microglial activation at the injury site compared to the saline infusion following mTBI. However, neither ssr-CHIMERA nor ketamine infusion altered synaptic density in the LGN, the brain region that receives axonal inputs through the OT. To our knowledge, this is the first study reporting the effects of ssr-CHIMERA, a recent mTBI paradigm, combined with IV ketamine infusion on synaptic density in experimental animals.

Several studies have demonstrated microglial activation in the cortex following mTBI. Repeated closed-head concussive injury in mice increased the numbers of activated microglia with shorter, thicker processes in the medial cortex (injury site) when measured at 42 days after the injury [31]. Similarly, repeated weight-drop injuries in rats increased microglial activation in the cortical injury site with an increased ratio of activated microglia with retracted processes to inactive microglia with extended processes at 24 h and 12 weeks post-injury [33]. After mild lateral fluid percussion injury (LFP), microglia with ameboid morphology were upregulated in the perilesional cortex directly underneath the impact site in rats [32]. Interestingly, some studies suggest that ameboid microglial activation is dependent on injury severity, with closed-head injury inducing hypertrophic microglia in the corpus callosum at smaller impact depths and ameboid microglia at larger impact depths in mice [54]. This is consistent with the literature suggesting that some activated states (i.e., hypertrophic microglia) may be observed with mild TBI, but ameboid microglia generally emerge with more severe TBI [30]. However, heterogeneity in microglial activation may exist between studies due to experimental conditions such as the mTBI model, which may affect the distribution and extent of microglial activation, quantification methods for microglial morphology, and interval between successive injuries. Nevertheless, findings from the current study are consistent with the literature reporting upregulation of morphologically active microglia after mTBI. 

Interestingly, a subanesthetic ketamine (10 mg/kg) infusion produced a greater activation of microglia than the saline infusion after the injury. To our knowledge, there is only one study reporting the effects of ketamine on microglial morphology following experimental TBI [55]. This study found that a high-dose ketamine–xylazine injection prior to a blast injury reduced hypertrophic (activated) microglia in the hippocampus of mice. The discrepancy between the previous and current studies may be due to different species (mice vs. rats), TBI models (blast vs. closed-head injury), and dose and route of ketamine administration. It is worth noting that microglia have dual functions following injury and inflammation. Microglia can be activated into a pro-damage (M1) state with harmful effects such as the release of neurotoxic and pro-inflammatory substances or a pro-repair (M2) state with beneficial effects such as clearing of cellular debris and release of neurotrophic factors and anti-inflammatory cytokines, depending on environmental influences [28]. These roles may also be influenced by injury severity, where mild injury with salvageable neurons may induce microglia to perform restorative, rather than neurotoxic, functions [56]. Given the fact that ketamine can shift hippocampal microglia toward an anti-inflammatory activation state as evidenced by the downregulation of M1 markers and upregulation of M2 markers following LPS treatment [57], the increased microglial activation at the injury site following ketamine infusion seen in the current study may be beneficial. Because subanesthetic doses of ketamine have putative neuroprotective and immunomodulatory effects, microglial activation following mTBI may be a recovery process mediated by ketamine.

White-matter damage, particularly after injuries involving rapid acceleration and deceleration, is a hallmark of TBI and may be implicated in consequences such as loss of consciousness [35,36]. The CHIMERA model has consistently produced axonal injury in the OT of experimental animals [7,11,16,17,42,43]. The OT conveys visual inputs from the RGCs to the LGN [44] and damage to RGCs resulted in degeneration in subsequent regions of the visual pathway, including the LGN [47,58,59]. Thus, we hypothesized that axonal injury in the OT may result in reduced synaptic density in the LGN. However, synaptic density in the LGN was not altered following ssr-CHIMERA at PID-4. In line with the current results, a repetitive mild weight-drop injury in mice caused axonal injury in the OT but did not produce synaptic density changes in the LGN as measured by synaptophysin staining [51]. Although conclusive evidence is still lacking, these results suggest that mTBI does not necessarily produce synaptic degeneration in the LGN despite producing axonal injury in the OT of brains.

There are possible reasons for the lack of synaptic density changes in the LGN after mTBI. One possibility is that the OT is more vulnerable to damage than the LGN. When both the OT and LGN were examined for neurodegeneration in a study using Fluoro-Jade C staining, only the OT, not the LGN, showed neurodegeneration [51]. Thus, the threshold of injury to cause significant damage to the LGN may be higher than for the OT, which may explain the negative results in the LGN. Additionally, due to geometric and structural properties, white-matter regions are particularly vulnerable to damage after mTBI [35,41]. Most OT synapses are with the LGN, but RGCs comprise only about 5–10% of LGN synapses [44]. The other inputs to the LGN come from the visual cortex and the brainstem [60]. This suggests that the OT may provide a minor contribution to LGN synapse density, so axonal damage in the OT may not necessarily affect overall synapse density in the LGN. Another possibility is that it may take longer to observe synaptic density loss in the LGN than to observe axonal injury in the OT. Thus, a future study is necessary to determine the long-term effects of mTBI on synaptic density in the LGN.

The rodent CHIMERA paradigm may be clinically relevant as it does not require a craniotomy or head restraint, which are invasive and not reflective of how humans sustain mTBI. Specifically, the ssr-CHIMERA is a reliable rodent model of mTBI based on microglial activation at the impact site without producing any cortical tissue loss in injured animals. A model with multiple concussive hits can induce sufficient cumulative damage while producing relatively low mortality rates [61]. Immediately consecutive hits replicate settings such as sports or combat where multiple concussions may be sustained within a short time frame. The short time between impacts may contribute to second-impact syndrome, where sustaining a second concussion before recovering from the first leads to serious and sometimes fatal consequences [62]. In addition to the clinical relevance of CHIMERA and its sequelae, the ketamine paradigm is also clinically relevant as ketamine is usually administered intravenously to patients. The same dose (10 mg/kg) of ketamine can produce opposite effects on fear memory depending on the route of administration [26], and the IV infusion is significantly more bioavailable than the IP bolus injection [63,64]. Therefore, the current study utilized a clinically relevant mTBI paradigm and drug administration regimen with well-established neuropathology biomarkers that allow for greater clinical translation.

Previous studies used conventional methods such as manual counting of dendrites and plasticity-related protein quantification to determine synaptic plasticity. Alternatively, the SEQUIN workflow utilizes super-resolution confocal scanning microscopy and is time-efficient compared to the conventional Golgi staining when quantifying synaptic density, with an image acquisition and analysis pipeline that can be largely automated [52,53]. SEQUIN allows investigators to define the synapse size as a particular distance between pre- and post-synaptic puncta, which addresses the lack of spatial information from Western blot analysis. However, SEQUIN is not perfect, with pitfalls including limited information on the physiology and ultrastructure of synapses, as well as the possibility of random (non-synaptic) puncta co-localizations, as previously described [53]. Despite these limitations, SEQUIN can be modified to improve its efficiency, resolution, and ease of use over conventional methods and presents a novel way forward in the quantification of synaptic density. Previous studies have indicated that mTBI reduced synaptic density measures [48,49,50], and ketamine’s mechanism of action includes synaptogenesis and synaptic plasticity [8]. Therefore, having an efficient and robust means to quantify synaptic density, rather than measuring pre- and post-synaptic markers in isolation, will allow investigators to conduct a thorough examination of molecular mechanisms.

The current study is not without limitations. We examined synaptic density in the LGN at one time point after mTBI. A two-photon imaging study in mice revealed that ketamine influenced spine formation with the growth of nascent spines that disappeared within four days [65]. The four-day time point is significant as spines that survive to this point become persistent and form functional synapses [66]. However, synaptic density and spine growth are known to be dynamic, and thus, multiple time points are necessary to capture the full picture of mTBI and ketamine. For example, hippocampal CA3 spine density was increased at 24 h but decreased at 7 days after ketamine (10 mg/kg) administration in male mice [67]. Second, the current study used only male rats. Ketamine is shown to produce sex-specific effects on various behaviors and brain functions [68,69], and this extends to synaptic plasticity as well [67,70]. Ketamine produced brain region-specific increases in spine density and synaptic protein levels in the hippocampus and medial prefrontal cortex (mPFC) with greater efficacy in male mice than female mice [67]. Another study reported that ketamine injection (5 mg/kg) after isolation stress rescued spine density loss in the mPFC and restored levels of synapsin-1 and PSD-95 only in male rats [70]. Thus, future studies should include both male and female rats for a more comprehensive study of synaptic plasticity following mTBI.

The rat CHIMERA paradigm produced mTBI phenotypes that are relevant to human mTBI. Morphological changes in microglia occur both in humans [15] and rodents [31,32,33]. The robust activation of microglia by ketamine in CHIMERA-injured animals may suggest the neuroprotective effects of ketamine given the dual role of microglia following injury. Moreover, white-matter injury is a hallmark of mTBI, which is commonly observed in various brain regions in humans [14,35,37,38,39,40]. In the current study, axonal injury occurred in the OT, which has been consistently observed in previous rodent mTBI studies [7,11,16,17,42,43]. However, synaptic density in the LGN, a region that receives axonal inputs through the OT, was not altered following CHIMERA injury in the early post-injury period. Overall, the current study demonstrates the feasibility of investigating mTBI mechanisms by combining a clinically relevant rodent mTBI model and IV ketamine infusion along with super-resolution confocal microscopy with efficient data analysis workflow.

## 4. Methods

### 4.1. Animals

Male Sprague–Dawley rats (9 weeks old) were purchased with jugular venous catheters surgically implanted at Envigo Laboratories (Dublin, VA, USA). Following three days of acclimation, animals were randomly assigned to one of the four groups, sham-saline, sham-ketamine, CHIMERA-saline, and CHIMERA-ketamine, using a 2 × 2 factorial design (N = 9–10 per group). This research protocol was approved by the Uniformed Services University IACUC (PSY-21-057) following all applicable federal regulations governing the protection of animals in research.

### 4.2. CHIMERA and Ketamine Infusion

Rats were anesthetized with isoflurane (5% for induction and 3% for maintenance via a nose cone) mixed with 100% oxygen before the CHIMERA injury. Each animal was placed in a dorsal position in the CHIMERA device with adhesive straps holding the body on the platform. The head was centered over cross-hairs on an aluminum plate, aligning the impact piston approximately on the bregma. A hole in the plate allowed a 100 g piston to impact the head (1.5 J, 5.5 m/s velocity). CHIMERA animals received three impacts (5–10 s apart) in a single injury session. Sham animals underwent the same procedure except for actual impacts to the head. After the injury, animals were returned to their home cages. Rats had a choice to drink acetaminophen water (1 mg/mL) for one day for post-injury analgesia. The total acetaminophen water intake was recorded and compared across the groups. One hour after the CHIMERA or sham injury, animals received either a saline vehicle or (*R*,*S*)-ketamine (10 mg/kg, IV) infusion over a 2 h period. The 10 mg/kg infusion dose produces analgesia without severe dissociation [25] and anti-inflammatory effects [7]. Animals received a bolus of IV ketamine (1 mg/kg) or saline before placement in the infusion chambers (Med Associates Inc., St. Albans, VT, USA), which were equipped with infusion pumps (Harvard Pump 11 Elite, Holliston, MA, USA). The tether system allowed free movement of the animals in the chambers during the ketamine infusion. Each chamber was equipped with two infrared photobeams to measure real-time locomotor activity via photobeam breaks. After the infusion, animals were returned to their home cages. A single ketamine infusion paradigm was used because animals sustained repetitive CHIMERA within a single injury session on the same day.

### 4.3. Brain Tissue Collection

Four days after the injury, rats were deeply anesthetized with isoflurane, and once unresponsive to paw pinch, a midline thoracotomy was performed to expose the heart. A trans-cardiac perfusion with ice-cold phosphate-buffered saline (PBS) and 10% neutral-buffered formalin was delivered through a peristaltic perfusion pump until adequate fixation was reached. The brain tissue was removed from the calvarium, post-fixed in 10% neutral-buffered formalin, and cryoprotected with 20% sucrose solution in PBS for three days before being frozen in dry ice and stored at −70 °C. Brains were sectioned with a sliding frozen microtome (Lecia Biosystems, Nußloch, Germany), and sections (40 μm) were stored in cryoprotectant solution at −20 °C.

### 4.4. Silver Staining

Silver staining was performed in the OT using the FD NeuroSilver Kit II (FD Neurotechnologies, Columbia, MD, USA) as described previously [7]. Briefly, free-floating sections were stored in 10% neutral-buffered formalin for one week, then washed in ddH_2_O twice before incubating in each mixture of reagents. After final incubation, sections were mounted on slides and dried overnight. The next day, they were cleared in xylene before being cover-slipped with Permount mounting medium (Fisher Chemical, Waltham, MA, USA). The OT region was imaged and scored for the intensity of silver staining on a scale from 0 to 3. A score of 0 indicated an absence of silver uptake, and scores of 1–3 indicated the presence of silver uptake, with 1 indicating minor, 2 indicating moderate, and 3 indicating extensive uptake, as a semi-quantitative measure for degenerating nerve fibers.

### 4.5. Iba-1 Immunohistochemistry

Iba-1 staining was performed in the cerebral cortex as described previously [7]. Briefly, free-floating brain sections were washed in tris-buffered saline with 0.05% triton (TBS-T), processed with 0.3% hydrogen peroxide for 30 min, washed, and incubated with blocking buffer (TBS-T with 0.2% Triton X-100, 10% goat serum (Vector Laboratories, Burlingame, CA, USA, S-1000), and 0.02% bovine serum albumin (Fisher Scientific, Waltham, MA, USA, Fraction V) for one hour. Primary antibodies of ionized calcium-binding adaptor molecule 1 (Iba-1) for microglia (Fujifilm Wako, Osaka, Japan, REF: 019-19741) were diluted 1:1000 in blocking buffer, and sections were incubated overnight at 4 °C. The following day, sections were washed and incubated in a secondary antibody solution (biotin-SP-conjugated goat anti-rabbit, Jackson Immunoresearch, West Grove, PA, USA) diluted 1:300 in blocking buffer for one hour. After washing, sections were incubated in ABC solution (Vectastain ABC Peroxidase kit, Vector Laboratories, Burlingame, CA, USA, REF: PK-4000) for 45 min, washed, and developed with a DAB peroxidase substrate kit (with nickel) and 3,3′-diaminobenzidine staining solution (Vector Laboratories, Burlingame, CA, USA, REF: SK-4100) for three minutes. Sections were put in phosphate buffer (PB) to stop the DAB reaction and mounted onto glass slides to dry overnight. Slides were then dehydrated in ethanol gradients (75–100%), cleared in xylene, and cover slipped with Permount mounting medium (Fisher Chemical, Waltham, MA, USA). Due to the nature of mild closed-head injury with rotational acceleration, cortical injury sites (size and exact location) were variable across CHIMERA animals. The observed cortical injury sites were anterior to the bregma area, which were determined based on microscopic examination of brain tissue sections. Activated microglia were identified based on the morphology of cells such as an ameboid (rounded cells with few to no processes) shape [71]. In contrast, inactive microglia are ramified with extensive, thin processes. This approach of a dichotomy of activated (ameboid) and inactive (ramified) microglia analyses has been used previously [72,73,74,75]. The injury site with activated microglia was traced as a region of interest (ROI), and the number of activated microglia was counted using the cell counter plugin in ImageJ software (version 1.53t). To minimize variability in the sizes of injury sites across CHIMERA animals, the same ROI was used to count activated microglia in the sub-cortical region (below the injury site) as a reference region. The ratio of activated microglia between the cortical injury site and sub-cortical reference region was calculated. The use of a reference region is similar to a previous study [76]. The Iba-1 staining for active microglia was scored by two experimenters blind to the treatment condition, and the scores were averaged for data analysis.

### 4.6. Synapsin and PSD-95 Immunohistochemistry

The LGN was determined based on the bregma coordinates from the rat brain atlas [77]. Four brain sections containing LGN from each animal were used for brain tissue analyses to capture representative data from the LGN. Immunofluorescent labeling was carried out as described previously [52]. Tissue sections were gradually warmed at room temperature, then washed three times for five minutes each with PBS using a six-well plate placed on a shaker. After the final wash, samples were placed in a six-well plate filled with a blocking buffer of 20% normal goat serum (Vector Laboratories, Newark, CA, USA) diluted in PBS for one hour. After blocking, sections were incubated in a primary antibody solution consisting of rabbit anti-PSD-95 (Invitrogen, Waltham, MA, USA) and guinea pig anti-Synapsin 1/2 (Synaptic Systems, Göttingen, Germany) diluted 1:1000 in 10% normal goat serum plus 0.3% Triton X-100 (Dow Chemical, Midland, MI, USA) for one day at 4 °C. After the primary antibody incubation, the sections were washed as described above, then incubated in a secondary antibody solution in 10% normal goat serum plus 0.3% Triton X-100 for four hours at room temperature. Brain sections were washed, mounted on clean microscope slides, and dried in a flat, dark location for approximately 10 min. While the samples were drying, mounting media were prepared by mixing AF300 and MWL4-88 (Electron Microscopy Sciences, Hatfield, PA, USA) in a 1:9 ratio, followed by vortexing and desktop centrifuging to remove air bubbles (5 min, 4000 rpm). Once the sections were dry, mounting medium was placed on the sample, and then the sample was cover slipped with spotless high-precision 1.5H coverslip glass (Marienfield, Lauda-Königshofen, Germany). Prepared slides were protected from light and cured at room temperature for 3–7 days before confocal microscope imaging.

### 4.7. SEQUIN

Sections were imaged on a Zeiss LSM 980 confocal microscope equipped with Airyscan 2 (Zeiss Group, Oberkochen, Germany). Scan parameters were established using previously described SEQUIN techniques [52]. The LGN was targeted under 10×power using an epiflourescent light source and microscope oculars on brain slices that contain the CA1 hippocampal region because LGN and CA1 are on the same coronal sections. Once the LGN was targeted, Zeiss 518 immersion oil was applied, and the area was re-targeted under 63× power. Once the final area was confirmed, confocal images were obtained via previously described experimental parameters [52]. Confocal microscope images were analyzed using Imaris software (version 10.0.1, Abingdon, United Kingdom). Images were converted to Imaris format and used for spot analysis for PSD-95 and synapsin puncta. Source channels were set to 488 nm and 594 nm for synapsin and PSD-95, respectively. The estimated XY diameter was set at 0.3 μm based on manual measurement of typical puncta size. Point spread elongation was set at 0.65 μm to account for the axial distortion of confocal-rendered images. Background subtraction and quality filters were utilized to capture maximum puncta detection while eliminating signal noise. Parameters were saved for batch analysis across all samples. After spot analysis, presynaptic to postsynaptic puncta analysis was determined using Imaris embedded software (version 10.0.1). Imaris identified and quantified post-synaptic PSD-95 puncta center points with a Euclidean distance < 0.55 μm from pre-synaptic synapsin puncta center points. Identified puncta pairs were divided by the scanned image volume to determine the relative synaptic density within each volume of sample analyzed. Densities from the four brain sections (one LGN per section) were averaged.

### 4.8. Statistical Analyses

Data were analyzed with a two-way ANOVA (CHIMERA × ketamine) followed by post hoc tests (Holm–Sidak multiple comparisons tests). For microglia data in CHIMERA injury, Student’s *t*-test was used to compare saline and ketamine groups. All data analyses and plotting graphs were performed with GraphPad Prism software (ver. 9.5.1). Significance is pre-determined at *p* < 0.05.

## Data Availability

Data sharing will be available upon request to the corresponding author.
